# Comparison of Shade Matching Ability among Dental Students under Different Lighting Conditions: A Cross-Sectional Study

**DOI:** 10.3390/ijerph191911892

**Published:** 2022-09-20

**Authors:** Rizwan Jouhar

**Affiliations:** Department of Restorative Dental Sciences, College of Dentistry, King Faisal University, Al-Ahsa 31982, Saudi Arabia; rjouhar@kfu.edu.sa; Tel.: +966-59-3114621

**Keywords:** tooth shade selection, dental student, knowledge, light source, shade matching

## Abstract

Successful esthetic dentistry to meet patient satisfaction and produce a progressive impact on someone’s personality cannot be succeeded without proper shade selection, both for direct or indirect restoration of dentition. The accurate shade selection is one of the most interesting phases of restoring the natural look of teeth. In clinical practices, dental students should be aware of the various light sources used for shade selection. The purpose of this research is to compare the shade matching ability of clinical and non-clinical students under clinical and correcting light. This comparative cross-sectional study was instigated amongst clinical and non-clinical students of the dental complex of King Faisal University, Kingdom of Saudi Arabia, after obtaining ethical approval from the Research Ethics Committee with reference number (KFU-REC-2022-MAR-EA000518). A total of 102 students assessed the shade under clinical (fluorescent light) and correcting light (handheld Dental Base Light) by using VITA Classical shade guides. Statistical analysis was done using SPSS version 23 (Armonk, NY, USA). The Chi-square test and *t*-test were used to evaluate the association between shade matching scores under correcting and clinical light. Out of 102 students, 41 (40.2%) were non-clinical and 61 (59.8%) were clinical, with a mean age of 21.66 ± 1.397 years. Shade matching scores were found significantly higher (*p* < 0.001) with the light-correcting device (2.29 ± 1.26) as compared to clinical light (1 ± 1.11) for non-clinical students. Similarly, clinical students also had significantly better (*p* < 0.001) shade matching with the light-correcting device (4.01 ± 1.34) in comparison to clinical light (2.47 ± 1.25). This study concluded that the shade matching scores under correcting light was significantly better than the results obtained by dental operatory light. Furthermore, it was also evidenced that clinical students’ skills in matching shades were significantly improved under a correcting light source as compared to non-clinical students.

## 1. Introduction

Accurate color matching in direct or indirect restorations is an imperative feature of prosthetic treatment that generally improves patient satisfaction [1]. Tooth color is frequently evaluated visually by the used shade guides that have already been provided by the manufacturers of dental products [2]. The human eye perceives color that may be diverted by light settings, gingival shade, and the colors of neighboring surroundings [3]. Tooth color comprises layers of enamel and dentine, which absorb, replicate, diffuse, or divert incident light, and makes the color quality [4]. The most critical influencing element in the choice of accurate shades is considered as light quality; variations in lighting conditions lead to alterations in the observed color [5]. Consequently, correct and reproducible color matching needs an accurate light source and spectral dispersion [5].

Generally, visual shade selection using a readymade shade guide is the most common technique used in clinical settings [6,7]. However, it is related to a high degree of partiality [8]. Though, this technique is considered subjective, as it is affected by age, gender, observer expertise, eye fatigue, and ambient lighting condition [9]. Additionally, many factors have an influence on shade selection, excluding eye fatigue, for instance, deficient color vision, light of surroundings, observer skills, tooth contour, and surface texture. Several studies supported that proficient skills in esthetic dentistry enhance the shade-matching capability, whereas some studies indicated that experience is not a significant aspect in shade matching [10]. Despite the acceptance of visual shade matching by shade guides, instrumental approaches have gained admiration, though they are costly and not always accessible to dental practitioners [11]. Shade selection by instrumental methods involves spectrophotometers, cross-polarizing filters, scanners, digital cameras, and smartphones [12]. Even though the instrumental approaches may assist the dentist, nonetheless, they cannot resolve all the difficulties involved in the shade-taking process. Consequently, the shade-matching process is frequently executed by comparing remaining tooth shades with a commercially existing shade guide [13].

The multiple elements that affect artificial tooth shade matching are the source of light, the object perceived, and the viewer skills [14]. For shade selection, three sources of light are used in dentistry: natural daytime light which is extremely variable; the operatory light of a dental unit, which reflects partiality to the red area of the perceptible spectrum than natural daylight; and lastly, fluorescent ceiling lights that have numerous color interpreting properties reliant on the definite color temperature [15]. The optimum state for tooth shade selection is indicated by a specified light with a color temperature in the middle of 5500 K and 6500 K with a Color Rendering Index (CRI) of more than 90. It is stated that the use of more than one light for the selection of shade of artificial teeth in prosthetics may encourage metamerism that is caused by intersecting lighting from different sources [15]. 

The lights used in the dental settings differ greatly with respect to the moment of the day, year, and category of light sources in the dental clinic resulting in a combination that develops between daylight and incandescent or fluorescent light [9]. In order to minimize the influence of environmental lighting for matching dental shades, color-corrected lighting tubes and light-correcting devices held in hand have been suggested [16]. Initially, fluorescent tubes were used as the main types of handheld light-correcting devices and had some problems; for instance, being bigger in size and difficult to handle. Therefore, the recognition of a novel generation of light-correcting devices that are more comprehensible and adaptable has been introduced. Several studies have revealed their competency in color-matching outcomes [17].

One study conducted in the United Kingdom compared the success of matching dental shades in a combination of color-correcting devices with a digital recording device as compared to a digital device alone in a normal lighting condition [18]. Similarly, another research revealed that a standard daylight lamp considerably increases the capability of matching shades in contrast to natural daylight [19]. It is evidently supported by another research that shade matching performance in corrected light sources was substantially improved as compared to natural or clinical light [20]. Even a low color-temperature light source remarkably increases color matching in deficient color vision persons [9]. Likewise, another study showed that generally, a light-correcting device makes the most favorable situation for enhancing shade matching [21]. Preferably, dental practitioners and technicians both should work in a comparable balanced, full-spectrum lighting environment in order to create closely adjusted spectral reflectance curves (optical properties) of natural and restoration, resulting in an admirable color match along with insignificant metamerism [15].

It is also believed that a bright background should not be positioned behind the observed teeth during shade assessment, as the dark oral cavity absorbs light and can affect shade selection [22]. Furthermore, it is also imperative to consider that the teeth observed in close proximity seem big and brighter [22]. Moreover, prosthodontists can match the color of teeth accurately as compared to general dental practitioners, as educational status and training in color selection can affect the matching of the optimum shade [23]. Conversely, there has been better responsiveness for aesthetically attractive restorations among patients. Accordingly, it is a practitioner’s concern to present restorations that can satisfactorily simulate the neighboring natural dentition [24]. 

The significance of color appearance in esthetic dentistry indicates the necessity of correct, high-quality shade matching. There is scarce scientific evidence available in comparing variances in visual shade matching using different devices; this matter remains debatable among researchers. Therefore, the purpose of the present study was to compare the accuracy of visual shade selection under different light sources such as dental operatory light and Dental Base Light of 5500°K color temperature among clinical and non-clinical students. The null hypothesis is that there is no effect of light source and year of dental schooling on tooth shade selection. 

## 2. Materials and Methods

This comparative cross-sectional study was instigated among clinical and non-clinical students of the dental complex of King Faisal University, Kingdom of Saudi Arabia after obtaining ethical approval from the Research Ethics Committee of King Faisal University with reference number (KFU-REC-2022-MAR-EA000518). Clinical students (year 4, 5, and year 6) were those attending dental clinics complex and treating patients under supervision, while non-clinical students (year 2, and year 3) were not treating dental patients. The sample was collected by using the convenience-sampling technique. A total of 102 male dental students (61 clinical and 41 non-clinical) showed normal color vision by successfully carrying out the Ishihara Color Blindness Test (24 Plate version, computer-based) [25] were included in this study, whereas students with deficient color vision were excluded from the study. None of the students was found colorblind, hence, there was no exclusion. Prior to registration, every student received accurate details on the study rules and signed the written permission.

### 2.1. Shade Guide Preparation

Four VITA Classical shade guides were used for the shade-matching procedure. Out of four shade guides, two were modified to fix randomly designated six shade tabs that consist of D3, C2, B3, A4, B2, and A2. The recognition of shade tabs was covered and assigned the numbers 1, 2, 3, 4, 5, and 6, respectively.

**Figure 1:**
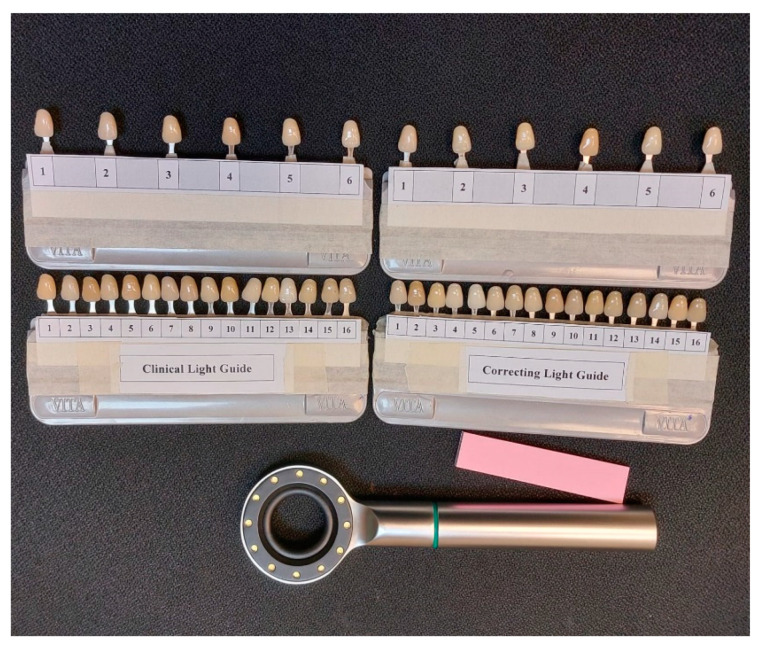
VITA Classical shade guides: Clinical Light Guide, and Correcting Light Guide.

In the two leftover shade guides, 14 shades were randomly organized with covered identification codes. Every tab was labeled with numbers from 1 to 14. As the 14 shades were randomly settled, each shade guide was identified for a specific lighting condition, i.e., Clinical Light Guide and Correcting Light Guide. Students were requested to match a single shade at a time with a specified shade guide for the different light sources, as shown in Figure 1. Six minutes were given for this process. Time was limited as extra time enhances the chances of error [20]. 

### 2.2. Shade Matching Conditions

**Figure 2:**
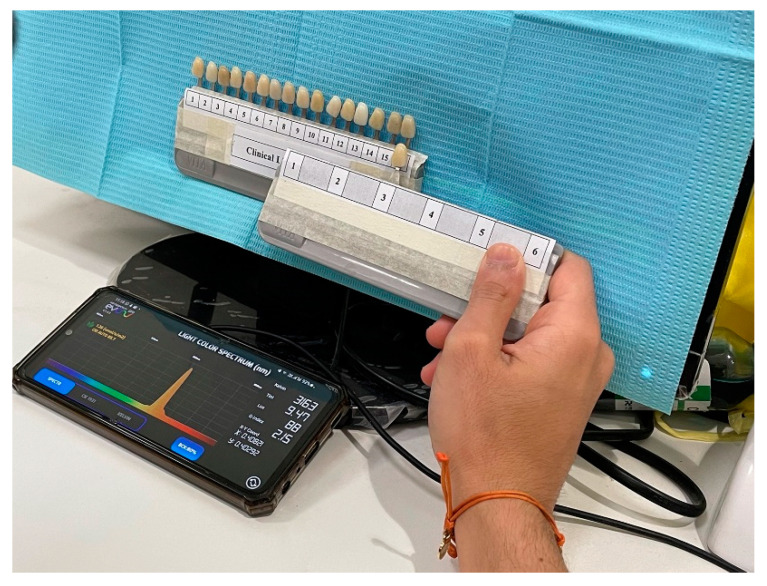
Dental operatory light (fluorescent light) with smartphone application Light Spectrum Pro EVO (AM Power Software, Via Località Passignano, 17 04025 Lenola (LT), Italia).

Two dental operatory lights were employed for shade selection; one with normal clinical lighting conditions and the other with correcting light. The dental operatory lighting condition consists of fluorescent light. The Color temperature of the dental operatory light was assessed by using the smartphone application Light Spectrum Pro EVO (AM Power Software, Via Località Passignano, 17 04025 Lenola (LT), Italia) was reported as 3400°K ± 150°K. Light Spectrum Pro EVO has an error of 2–8% as compared to present professional products in the market, as shown in Figure 2.

**Figure 3:**
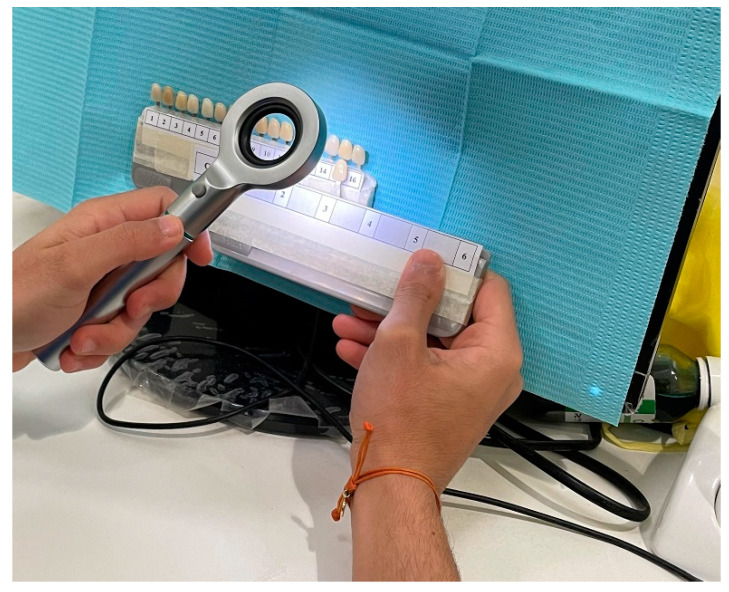
Correcting light with handheld Dental Base Light (Tri-Shade, Zhengzhou, China).

A distance of 10 cm from the shade tab was maintained in shade-matching under the correcting light that was executed by the use of handheld Dental Base Light (Tri-Shade, Zhengzhou, China). The Dental Base Light comprises integrated 12 LED technology. The appropriate color temperature selected from correcting light was 5500°K for shade selection as shown in Figure 3. 

The background was kept blue to eliminate color distraction and to reduce eye fatigue. Shade matching under clinical light and correcting light was performed during the daytime between the hours of 10:30 A.M. and 1:30 P.M. An interval of 1 day was maintained between sessions of shade selection as previous subjective background can adversely affect shade matching. After matching selected items (shade tabs with the identification code concealed) to a VITA Classical shade guide, the selected shade tabs number was documented (the highest score possible was 6) and the correct matches were calculated. Scores were calculated by adding the number of correct matches. The highest score was considered if matched for all the items successfully by the students. 

**Figure 4:**
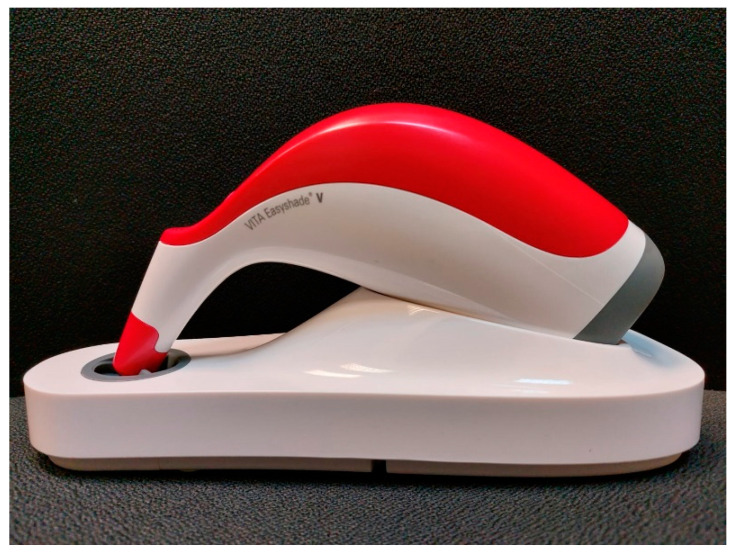
Vita Easyshade^®^ V, Bad Säckingen, Germany.

These selected shades under clinical and correcting light were confirmed by using spectrophotometers such as Vita Easy Shade^®^ V in order to reduce the possibility of human error and enhance the high level of reliability, as shown in Figure 4. 

### 2.3. Statistical Analysis

The data was statistically analyzed using SPSS version 25.0 (IBM Corp., Armonk, NY, USA). Categorical variables such as academic year, clinical level, age, and selected shades were documented as frequencies and percentages and mean ± SD. A Chi-square test was used to assess the association of shades with clinical and non-clinical students, and a *t*-test was applied to determine the association between shade matching scores and clinical and non-clinical students under clinical and correcting light. A *p*-value < 0.05 was considered as statistically significant.

## 3. Results

A total of 102 students participated in this study with a mean age of 21.66 ± 1.397 years. Among the study participants, 19 were from the 2nd year, 22 were from the 3rd year, 21 were from the 4th year, 19 were from the 5th year, and 21 were from the 6th year. None of the students withdraw from this study after participation. Concerning the clinical level, 41 (40.2%) were non-clinical and 61 (59.8%) were clinical students, as shown in Table 1. 

Comparison of shade selection under clinical and correcting light of non-clinical students revealed that shade D3 was the highest selected shade, followed by A4, A2, and B2 while C2 was the least selected shade of non-clinical students. A statistically significant difference was evident in shades D3, B3, B2, and A2 between clinical and correcting light for non-clinical students (*p* < 0.05). On the other hand, an insignificant difference was found in C2 and A4 shades between clinical and correcting light for non-clinical students (*p* > 0.05). A comparison of shade selection under clinical and correcting light of clinical students revealed that a statistically significant difference was evident in shades D3, B3, C2, A4, and A2 under clinical and correcting light (*p* < 0.05), and an insignificant difference was evident in B2 shade under clinical and correcting light (*p* > 0.05), as shown in Table 2.

A comparison of shade selection of non-clinical and clinical students under clinical light revealed that D3 shades had the highest selected shade of non-clinical students, followed by A4 and B2, while C2 was the least selected shade. A statistically significant difference was seen in shades D3, B3, B2, C2, and A2 between clinical and non-clinical students (*p* < 0.05). On the other hand, an insignificant difference was found in the A4 shade between clinical and non-clinical students (*p* > 0.05). Furthermore, a comparison of shade selection of non-clinical and clinical students under correcting light revealed that D3 and A2 shades had the highest selected shades of non-clinical students, followed by A4 and B3, while C2 was the least selected shade. A statistically significant difference was seen in all shades between clinical and non-clinical students under correcting light (*p* < 0.05), as shown in Table 3.

Shade matching scores were found to be significantly higher with the light-correcting device than with clinical light 2.29 ± 1.26 and 1 ± 1.11, respectively, in non-clinical students and 4.01 ± 1.34 and 2.47 ± 1.25, respectively, in clinical students (*p* < 0.001), as shown in Table 4.

## 4. Discussion

The present study was performed to compare the accuracy of visual shade selection under different light sources such as dental operatory light and with Dental Base Light of 5500°K color temperature among clinical and non-clinical students. All students were male with the mean age of 21.66 ± 1.397 years and having a different year of dental schooling and clinical experience. The result of the present study showed that changes in lighting conditions significantly improved the means score of shade selection in both clinical and non-clinical students (*p* < 0.001). Furthermore, clinical students’ shade matching ability was found significantly better (*p* < 0.05) under correcting light as compared to non-clinical students in all shades. 

The light source is an influencing element in shade matching. Even though natural daylight has been advocated to be the perfect light source for shade matching, but the quality of daylight is incompatible and impossible to select shades always during the daytime. Consequently, improvement in shade matching performance can be achieved by using a consistent light source along with a suitable environmental situation [20]. This study demonstrated the shade-matching ability of clinical and non-clinical students under correcting and clinical lighting conditions.

The current study revealed that shade matching scores were observed to be significantly higher with the light-correcting device in contrast with clinical light (*p* < 0.001). These results were endorsed by another study in which 165 male and 51 female students were involved, with a mean age of 26 years. The shade matching scores were highly significant with the light-correcting device as compared with natural light 11.4 ± 1.9 and 10.4 ± 2.0, respectively (*p* < 0.001), while gender and experience were not found to be the factors affecting shade matching [26].

Interestingly, the present study evaluated the shade selection under clinical and handheld correcting lighting conditions and showed a statistically significant difference between both lighting systems. These findings are in line with Mohammad et al. study, which showed that handheld stable light devices are inexpensive and applied form of balanced light that enhances the significance of shade matching environment [27]. These findings were also in agreement with Nakhei et al. who supported our outcomes that superior results were achieved in shade matching using a correcting light device in contrast with natural and clinical light [20]. Moreover, these results were also consistent with the research by Mete et al. [28], Curd et al. [26], and Corcodel et al. [19]. 

Similarly, further studies signified the importance of lighting conditions in shade matching. They revealed in their studies that the natural light condition is one of the most effective features in shade matching skills and this natural light condition can be achieved using the correcting light under a clinical setup for better shade selection [29,30,31]. Likewise, it has been stated that lighting conditions have an influence on the shade matching capability of color vision deficient individuals, and there is a remarkable perfection in their shade matching skills with low-temperature light sources [32,33]. The present study findings were consistent with the above-mentioned research and showed that shade matching performance was superior under correcting light over clinical lighting conditions. 

Several studies demonstrated that dental practitioners were unpredictable in color matching [34,35]. Multiple factors like age, level of expertise, eye fatigue, and physiological situations such as deficient color vision may cause partiality and discrepancies [36]. The result of the present study—that correcting light has a positive role in shade selection—is in line with Clary et al.’s study, wherein data was gathered only from 3rd-year dental students, but the result was the same [37], as it is believed that the majority of dental students are usually young adults having good physical health with no medical disorders that can affect their color matching proficiency [38]. Additionally, in Paravina et al. study, A2, A3, and A4 shades were selected owing to a higher frequency in the population of these shades (43%) and their variation in lightness (medium light, medium-dark, and dark) [39]. 

Ideally, shade matching should be executed in natural daylight settings [39]. However, it is difficult to achieve these conditions. Former research has revealed that shade selection using artificial color-corrected light gives improved results in comparison with natural light [40]. It is also thought that natural daylight shows variability and unreliability and should not be used as the control [41]. In the present study, both clinical and correcting light were used to select the shade. However, enhanced results were obtained under correcting light. There was a significant difference found in the mean scores of shade selection under correcting and clinical light. (*p* < 0.001). These observations were supported by another study in which handheld correcting lights were observed to be improved as compared to the control and revealed a statically significant enhancement between scores of 7.8 than 7.2, correspondingly [37]. It is due to the fact that observers were targeted merely and directly through a small frame of the handheld light during shade matching. Consequently, this might could have decreased background disruption and improved attentiveness on the shade-matching skills.

Educational level, along with training on shade matching, exhibits a significant role in shade matching accuracy. Earlier studies have indicated that to improve shade-matching performance, dental practitioners must be participated in practical courses, ongoing education strategies, and further teaching classes [42]. As far as the present study is concerned, a significant difference was evident between all shade tabs under correcting light among clinical and non-clinical students (*p* < 0.05) thereby proving clinical experience of students on color matching leads to enhancing the accuracy of shade selection. These findings were endorsed by another study and their data should encourage dental practitioners to integrate color-corrected light devices into their performances to select the shade, search for accurate training for shade matching, and actively participate in implementing their information [37]. Likewise, one additional study supported the present study and specified that the student’s shade matching skills under a color-correcting device enhanced shade selection as compared to the conventional technique in standard lighting conditions [18].

Color vision is a crucial element of restorative, prosthodontics, and aesthetic dentistry because deficient color vision leads to develop difficulties in observing accurate color in contrast to healthy color vision dentists [43,44]. According to one study, it was proposed that participants with any form of color vision deficiency should be excluded from studies as it had already been shown that color vision impairment may lead to inferior color matching quality [45]. These findings were endorsed by the present study that none of the students revealed any color vision impairments.

This result of this study should be seen under certain limitations that only shade tab matching has been considered, whereas further research is required on matching shade tabs with natural dentition and assessment by other shade measuring devices. Within the limitation of this study, using a usual light source with a full spectrum and offering suitable conditions in the clinical setting is suggested. Currently, synthetic lights are employed generally in most dental clinics, and also many dental practices are executed where daylight is frequently not available. Consequently, it is essential to allot a separate place for shade matching as unnecessary lighting conditions may adversely affect the shade matching technique. Furthermore, this study’s results cannot be generalized as the study was conducted only on male students with less clinical experience. 

Further studies are recommended to compare Dental Base Light (Tri-Shade, Zhengzhou, China) with other correcting light and to conduct large randomized controlled clinical trials, with the purpose of giving improved vision into the performance of numerous computerized methods to attain successful shade matching. Furthermore, spectrophotometers already exist in the market that require more research to explore these devices, which could offer a comprehensive understanding of shade matching between the tooth and the restoration.

## 5. Conclusions

This study concluded that the shade matching scores under correcting lights were significantly better than the results achieved by clinical lights. Furthermore, it was also observed that clinical and non-clinical students’ skills in matching shades were improved considerably under a correcting light source as compared to dental operatory light. Furthermore, clinical experience students were better than non-clinical students in shade selection.

## Figures and Tables

**Table 1:** Demographic characteristics of participants (*n* = 102).

	Age	Mean (Years)	SD (Years)
		21.66	1.397
	Academic Year	Frequency (*n* = 102)	Percentage (%)
**Non-Clinical Students** (*n* = 41)	2nd Year	19	18.6%
	3rd Year	22	21.6%
**Clinical Students** (*n* = 61)	4th Year	21	20.6%
	5th Year	19	18.6%
	6th Year	21	20.6%

**Table 2:** Comparison of shade selection of non-clinical students and clinical students under clinical and correcting light.

		D3	C2	B3	A4	B2	A2
		% (no.)	% (no.)	% (no.)	% (no.)	% (no.)	% (no.)
Non Clinical Students (*n* = 41)	**Clinical Light**	21.95 (9)	9.75 (4)	17.07 (7)	21.95 (9)	21.95 (5)	17.07 (7)
	**Correcting Light**	43.90 (18)	24.39 (10)	39.02 (16)	41.46 (17)	36.58 (15)	43.90 (18)
	***p*-value**	0.034	0.078	0.027	0.058	0.010	0.008
Clinical Students (*n* = 61)	**Clinical Light**	44.26 (27)	37.70 (23)	39.34 (24)	32.78 (20)	52.45 (32)	37.70 (23)
	**Correcting Light**	75.40 (46)	59.01 (36)	72.13 (44)	63.93 (39)	65.57 (40)	70.49 (43)
	***p*-value**	<0.001	0.019	0.000	0.001	0.141	<0.001

**Table 3:** Comparison of shade selection between non-clinical students and clinical students under clinical and correcting light.

		D3	C2	B3	A4	B2	A2
		% (no.)	% (no.)	% (no.)	% (no.)	% (no.)	% (no.)
Clinical Light	**Non Clinical Students (*n* = 41)**	21.95 (9)	9.75 (4)	17.07 (7)	21.95 (9)	21.95 (5)	17.07 (7)
	**Clinical Students (*n* = 61)**	44.26 (27)	37.70 (23)	39.34 (24)	32.78 (20)	52.45 (32)	37.70 (23)
	***p*-value**	0.021	0.002	0.016	0.234	<0.001	0.025
Correcting Light	**Non Clinical Students (*n* = 41)**	43.90 (18)	24.39 (10)	39.02 (16)	41.46 (17)	36.58 (15)	43.90 (18)
	**Clinical Students (*n* = 61)**	75.40 (46)	59.01 (36)	72.13 (44)	63.93 (39)	65.57 (40)	70.49 (43)
	***p*-value**	0.001	0.001	0.001	0.025	0.004	0.007

**Table 4:** Comparison of shade matching scores under clinical and correcting light.

Non-Clinical Students		
	**Mean ± SD**	***p*-Value**
Clinical light	1 ± 1.11	<0.001
Correcting Light	2.29 ± 1.26	
Clinical Students		
	**Mean ± SD**	***p*-Value**
Clinical light	2.47 ± 1.25	<0.001
Correcting Light	4.01 ± 1.34	

## Data Availability

The data presented in this study will be available on request from the corresponding author.
